# Prophylactic versus on‐demand transfusion in pregnant women with sickle cell disease: A systematic review and meta‐analysis of randomised controlled trials

**DOI:** 10.1002/jha2.1086

**Published:** 2025-04-24

**Authors:** Denise Menezes Brunetta, Evangelia Vlachodimitropoulou, Nita Prasannan, Paul T. Seed, Eugene Oteng‐Ntim

**Affiliations:** ^1^ Division of Women's Health Kings College London St Thomas’ Hospital London UK; ^2^ Empresa Brasileira de Servicos Hospitalares ‐ EBSERH Fortaleza Brazil; ^3^ Centro de Hematologia e Hemoterapia do Ceara ‐ HEMOCE Fortaleza Brazil; ^4^ Universidade Federal do Ceara Fortaleza Brazil; ^5^ Guy's and St Thomas’ NHS Foundation Trust London UK; ^6^ Department of Non‐communicable Disease Epidemiology London School of Hygiene and Tropical Medicine London UK

**Keywords:** pregnancy, randomised controlled trials, sickle cell disease, systematic review, transfusion

## Abstract

**Introduction:**

Sickle cell disease (SCD) poses significant risks during pregnancy. Transfusions are the only recommended treatment, but there is no strong evidence of its efficacy. The aim of this study was to evaluate prophylactic transfusion on pregnancy outcomes.

**Methods:**

We performed a systematic review and meta‐analysis (PROSPERO‐CRD42024510511), using MEDLINE, EMBASE, Cochrane, Web of Science, and Maternity and Infant Care. No date or language restrictions were applied. Inclusion criteria comprised randomised‐controlled trials (RCTs) involving SCD pregnancy, comparing maternal and foetal outcomes for prophylactic versus on‐demand transfusions. Two independent reviewers performed screening, selection, and data extraction, following PRISMA. Two authors independently assessed certainty and risk‐of‐bias. Data were pooled using random‐effects model. Primary outcomes included mortality, vaso‐occlusive crisis (VOC), acute chest syndrome, venous thromboembolism and preterm delivery. The measure of the effect was the unadjusted odds ratio (OR), calculated from numbers of events.

**Results:**

Ninety‐one studies were identified and two RCTs (106 patients) were included, with uncertain and low risk of bias. Prophylactic transfusions reduced VOC, OR of 0.197 (95% CI 0.08–0.49). However, due to the small number of patients, this meta‐analysis was underpowered to evaluate other outcomes.

**Conclusion:**

A larger RCT is needed to comprehensively assess the impact of prophylactic transfusion in SCD pregnancy.

## 1 INTRODUCTION

Sickle cell disease (SCD) encompasses a group of inherited haemolytic disorders characterised by the presence of haemoglobin S (HbS). HbS undergoes polymerisation in low‐oxygen conditions leading to the characteristic sickle shape of red blood cells (RBC). The most prevalent and severe form of SCD is the homozygous presentation, known as sickle cell anaemia (HbSS) [1]. Other forms of SCD occur when a different abnormal haemoglobin gene is coinherited. These include HbSC and HbSBthal, as well as less common genotypes like HbSD and HbSO_arab_ [1].

SCD ranks among the most widespread genetic disorders, with an estimated 515,000 infants born each year globally [2]. Due to increase in life expectancy and improved management of the condition, more women are reaching reproductive age. Thus, there is a growing focus on improving outcomes for pregnant women [3, 4].

The manifestation of SCD exhibits considerable variability, influenced by genetic modifiers, such as coinheritance of α‐thalassaemia or mutations affecting foetal Hb levels, even within the same genotype [1]. Despite the phenotypic variability, all pregnant women with SCD face high‐risk pregnancies, irrespective of geographic location or income level [5]. SCD is notably associated with elevated morbidity and mortality rates for both the mother and the foetus [3, 6, 7].

Pregnant women with SCD are at an increased risk of pregnancy‐related complications, including pre‐eclampsia and venous thromboembolism (VTE) [7]. SCD‐related morbidity may also increase during pregnancy, such as exacerbation of anaemia, vaso‐occlusive crisis (VOC) and acute chest syndrome (ACS) [3, 8]. Furthermore, SCD is also associated with adverse foetal/neonatal outcomes, characterised by a higher prevalence of small for gestational age (SGA) infants, prematurity, stillbirths and neonatal mortality [6, 8].

Since hydroxycarbamide and L‐glutamine are not recommended during gestation, RBC transfusion is the only treatment currently available for pregnant women with SCD [4]. Transfusion plays a crucial role in addressing many SCD complications [1]. However, it is associated with risks, including alloimmunisation, haemolytic transfusion reactions (HTR) and iron overload [9, 10]. Significant knowledge gaps persist regarding prophylactic transfusions, and this is an opportune moment to conduct a systematic review and meta‐analysis, particularly in light of the recently published randomised controlled trial (RCT) TAPS2 [11]. This study aims to clarify the risks and benefits of prophylactic transfusions in SCD pregnancy.

## 2 METHODS

### 2.1 Search strategy and selection criteria

This is a systematic review and meta‐analysis and adhered to the Preferred Reporting Items for Systematic Reviews and Meta‐Analyses (PRISMA) guidelines. Studies were included if they (1) were RCTs (2) involved pregnant women with SCD (HbSS and other genotypes); (3) compared maternal and foetal outcomes when top‐up (transfusion without prior blood withdrawal from the patient) or partial or full exchange transfusions (either manual or automated) were given prophylactically versus on‐demand. There were no date or language restrictions.

The following data sources were evaluated: MEDLINE, EMBASE, Cochrane, Web of Science, and Maternity and Infant Care. Literature was last consulted on 07 July 2024. The search terms were (sickle or haemoglobinopathy or haemoglobinopathy) AND (pregnant or pregnancy or maternal) AND (transfusion or exchange or erythrocytapheresis) AND (randomized or randomised or randomly).

Abstracts and full‐text articles were reviewed independently by two authors (EV and DMB) to identify those papers that met inclusion criteria.

### 2.2 Data Analysis

Data collection was carried out independently using a standard form from published reports. Grade score was used to assess certainty in the body of evidence for the outcomes.

The following information was extracted: title, authors, year of publication, period of the study, location, number of women in each arm, transfusion method (top‐up or manual or automated‐exchange transfusion), RBC characteristics (phenotype‐compatibility, leucoreduction), singleton or multiple gestations, previous obstetric history, and SCD genotype. Data were extracted on the following outcomes:

Maternal:

- Death
- VOC
- ACS
- VTE
- Caesarean delivery

Foetal and neonatal:

- Stillbirth
- Neonatal death
- Preterm delivery,
- Delivery of SGA infant (< 10^th^ centile) or intrauterine growth restriction (IUGR)

RBC transfusion:

- Transfusion reactions

Two review authors (EV and DMB) independently assessed the risk of bias for each study using the ROB‐2 tool. Any disagreement was resolved by a third assessor (EO).

The characteristics of each study were tabulated and compared against the planned group for each synthesis. Absolute numbers of successes and failures for each variable in each arm were tabulated. The main measure of the effect of prophylactic transfusion on maternal and foetal/neonatal outcomes was the unadjusted odds ratio (OR), calculated from the given numbers of events. In each analysis, the reference group was women submitted only to on‐demand transfusions during pregnancy. For all outcomes, analyses were carried out on an intention‐to‐treat basis.

We used statistical meta‐analysis with a random effects model to assess the association between transfusion and adverse pregnancy outcomes. The level of heterogeneity was expressed using τ^2^ (rather than I^2^), due to the small number of studies included. As with I^2^, a τ^2^ value of 0 indicates no unexplained variation, whereas values of τ^2^ ∼0.5 indicate a typical ratio of 2 between study estimates of the risk ratio; higher values indicate higher levels of variation. Unlike I^2^, there is no artificial upper limit of 100%.

All analyses were performed using Stata, version 18 (StataCorp, College Station) and the command Meta‐analysis. By default, Stata meta esize adds 0.5 to each cell of studies containing zero cells [12]. So, for a study reporting zero cells, the number of successes, *e*, was incremented by 0.5, the number of failures was incremented by 0.5, and therefore, the total sample size, *n*, was increased by 1. Tables and forest plots with reference lines were used to display results of individual studies and syntheses.

The study protocol was registered on PROSPERO (CRD42024510511).

## 3 RESULTS

**FIGURE 1:**
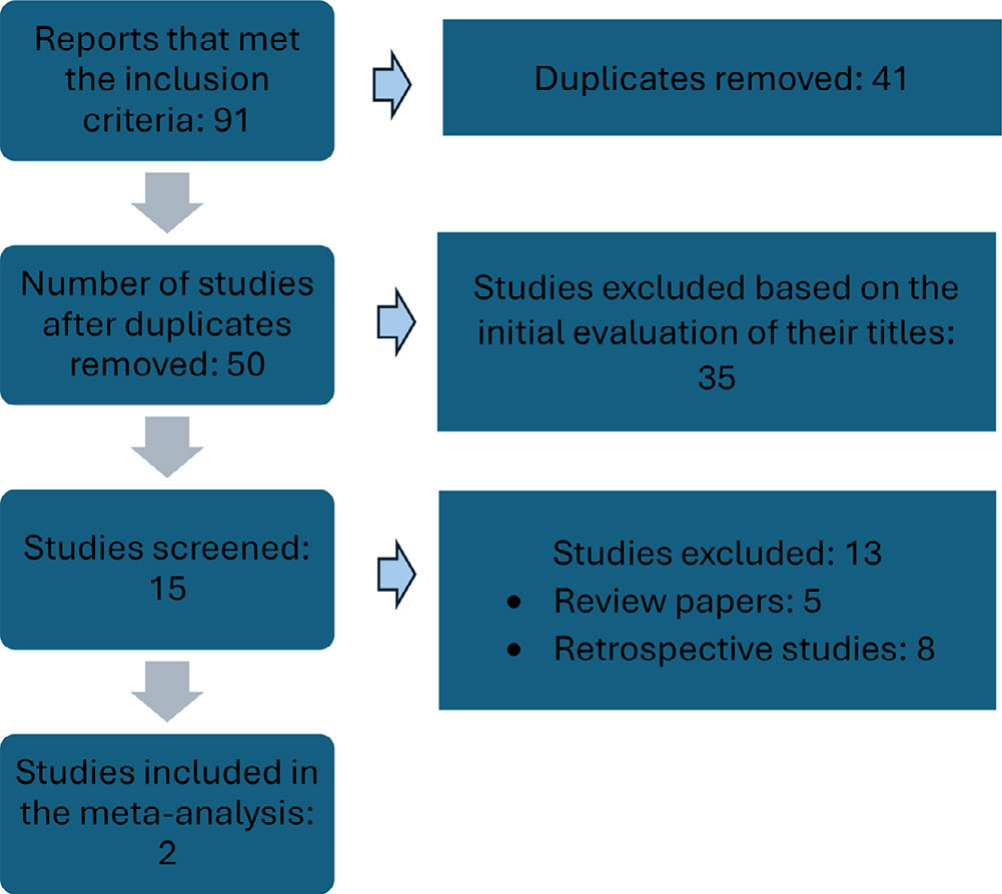
PRISMA flow diagram for identification and selection of articles for inclusion in the meta‐analysis. PRISMA, Preferred Reporting Items for Systematic reviews and Meta‐Analyses.

The search retrieved 91 studies, with 50 remaining after duplicates were excluded. From these, 35 were excluded based on the initial evaluation of their titles. The remaining 15 studies were reviewed through their abstracts or full papers. Only two studies met the inclusion criteria, Koshy et al. [13] and Oteng‐Ntim et al. [11]. The other studies were excluded because they were not RCTs, or the data did not pertain to the review of interest. The PRISMA flow diagram is shown in Figure 1.

The characteristics of the studies are described in Table 1. Table 2 presents the summary statistics for all the outcomes and the relative effect with 95% confidence interval (CI) of the synthesis.

**TABLE 1:** Studies characteristics.

Study	Koshy	Oteng‐Ntim
Period	January 1979 to March 1986	April 2019 to October 2022
Location	United States of America (7 centres)	England (7 centres)
Sickle genotype	HbSS	SS, SC or S+Other
Exclusion criteria	Neurologic dysfunction, nephrotic syndrome, chronic renal failure, persistent liver disease, chronic lung disease, coagulopathy, or the presence of multiple red‐cell antibodies that would make matching for blood groups difficult.	Patients unable or unwilling to give written informed consent; on long‐term transfusion program prior to pregnancy for the amelioration of sickle cell disease; unable to receive blood transfusion for social, clinical or religious reasons; current diagnosis of major medical or psychiatric comorbidity that, in the randomising clinicians’ opinion, rendered them unable to enter the trial; prior hyperhaemolysis; red cell phenotype or antibodies present that may prevent likely provision of adequate red cell units to support elective exchange‐transfusion program.
Gestation characteristics	<28 weeks, singleton and multiple gestations	Singleton pregnancy between 6+0 and 18+0 weeks’ gestation
Previous obstetric history	Stillbirths and neonatal deaths in some participants	Stillbirth in one participant and some participants with previous spontaneous miscarriages
Number in each group	36 prophylactic and 36 control	18 prophylactic and 17 control
Intervention in prophylactic group	Simple transfusion or manual partial exchange transfusion, repeated weekly.	Automated exchange blood transfusion
Goals in the prophylactic group	Maintain the haemoglobin concentration between 10 and 11 g per decilitre, or the haematocrit near 33%, and to reduce the level of HbS below 35%	Maintain HbS% below 30% or combined HbS and HbC below 30%
RBC characteristics	Washed frozen	Leucoreduced RBC
Phenotype compatibility	Not informed	Rh and K matched
Outcomes	Obstetric complications, sickle‐cell disease related complications, transfusion related complications (total units of blood transfused, blood transfusion reaction), perinatal outcomes	Recruitment rate (primary outcome), acceptability of the intervention, and retention. Secondary outcomes: Safety and maternal and infant clinical outcomes.

Abbreviations: Hb, haemoglobin; RBC, red blood cell.

**TABLE 2:** Summary of findings. Prophylactic red cell transfusion versus selective transfusion in pregnant women with sickle cell disease.

Outcomes	Effect estimates for each study (95% CI)		Relative effect (95% CI)	Number of participants (trials)	Quality of the evidence (GRADE)
	Koshy et al., 1988	Oteng‐Ntim et al., 2024			
Maternal mortality	No deaths occurred in either trial arm		‐	106 (2 RCTs)	⊕⊝⊝⊝ Very low^a^, ^b^, ^c^
Maternal complications: Vaso‐occlusive crisis	OR 0.161 (0.05–0.51)	OR 0.274 (0.06–1.19)	OR 0.197 (0.08–0.49)	106 (2 RCTs)	⊕⊕⊝⊝ Low^a^, ^c^
Maternal complications: Venous thromboembolism	OR 1.000 (0.02–51.76)	OR 3.182 (0.12–83.76)	OR 1.986 (0.16–24.64)	106 (2 RCTs)	⊕⊕⊝⊝ Low^a^, ^c^, ^d^
Maternal complications: Acute chest syndrome	OR 0.647 (0.10–4.12)	OR 0.31 (0.01–8.27)	OR 0.699 (0.11–2.72)	106 (2 RCTs)	⊕⊕⊝⊝ Low^c^, ^d^
Caesarean delivery	OR 0.743 (0.25–2.17)	OR 2.275 (0.52–9.99)	OR 1.152 (0.39–3.36)	106 (2 RCTs)	⊕⊕⊝⊝ Low^c^, ^d^
Preterm delivery	OR 3.182 (1.06–9.59)	OR 0.150 (0.01–1.46)	OR 0.818 (0.04–16.02)	106 (2 RCTs)	⊕⊝⊝⊝ Very low^b^, ^c^, ^d^
Foetal/neonatal outcome: Neonatal deaths	OR 5.000 (0.23–107.7)	OR 1.000 (0.02–53.28)	OR 2.741 (0.24–31.13)	110 (2 RCTs)	⊕⊕⊝⊝ Low^c^, ^d^
Foetal/neonatal outcome: Delivery of a small for gestational age infant/ Intrauterine growth restriction	OR 0.630 (0.18–2.2)	OR 1.000 (0.17–5.83)	OR 0.735 (0.26–2.04)	110 (2 RCTs)	⊕⊕⊝⊝ Low^c^, ^d^
Adverse events associated with transfusion: Alloimmunisation and delayed haemolytic transfusion reaction	OR 1.818 (0.69–4.78)	OR 1.000 (0.06–17.41)	OR 1.710 (0.68–4.27)	106 (2 RCTs)	⊕⊕⊝⊝ Low^c^, ^d^

*Note*: The risk in the intervention group (and its 95% confidence interval) is based on the assumed risk in the comparison group and the relative effect of the intervention (and its 95% CI).

Abbreviations: CI, confidence interval; OR, odds ratio; RCT, randomised controlled trial; RR, risk ratio.

GRADE Working Group grades of evidence High quality: We are very confident that the true effect lies close to that of the estimate of the effect. Moderate quality: We are moderately confident in the effect estimate: The true effect is likely to be close to the estimate of the effect, but there is a possibility that it is substantially different. Low quality: Our confidence in the effect estimate is limited: The true effect may be substantially different from the estimate of the effect. Very low quality: We have very little confidence in the effect estimate: The true effect is likely to be substantially different from the estimate of effect.

^a^ We downgraded the quality of the evidence by 1 due to risk of bias. Trial was not blinded, and the outcome assessor was the patient.

^b^ We downgraded the quality of the evidence by 1 due to inconsistency. Statistical analysis (τ^2^) suggests heterogeneity.

^c^ We downgraded the quality of the evidence by 1 due to indirectness. Koshy trial recruited only sickle cell anaemia (SS) patients.

^d^ We downgraded the quality of the evidence by 1 due to imprecision. Small number of participants and events and very wide CIs.

Koshy study did not include the definition for what they called “intrauterine growth retardation” [13]. Despite this, it was evaluated in this meta‐analysis together with delivery of small for gestational age infant (< 10th centile) as defined by Oteng‐Ntim [11].

**FIGURE 2:**
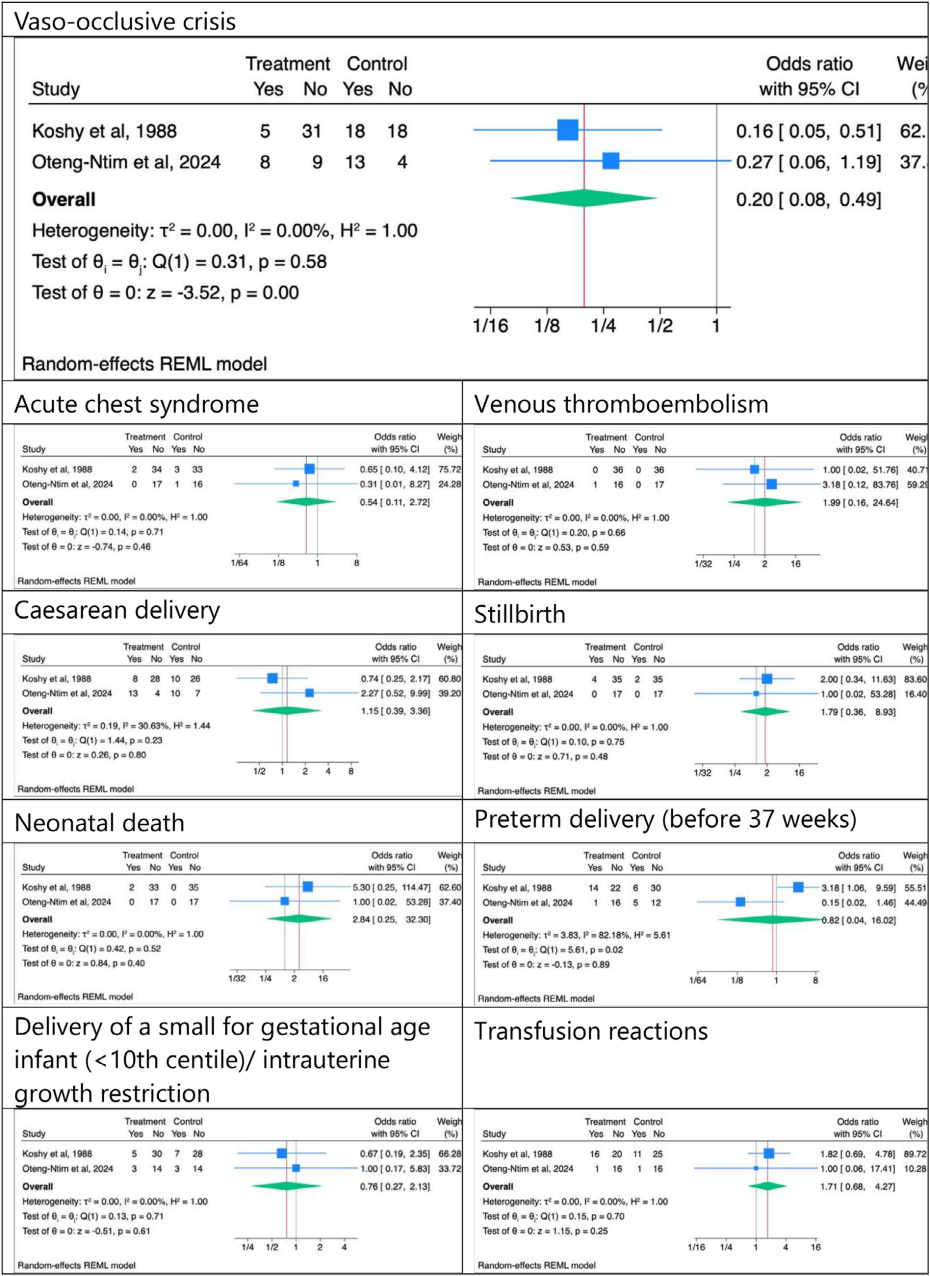
Random‐effects models of maternal and foetal outcomes. CI, Confidence interval.

**FIGURE 3:**
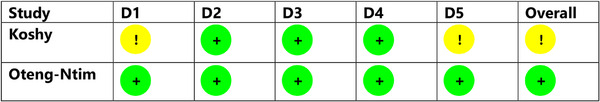
‘Risk of Bias’ summary: The review authors' judgements about each risk of bias item for each included study. D1: Randomisation process, D2: Deviations from the intended interventions, D3: Missing outcome data, D4: Measurement of the outcome, D5: Selection of the reported result. 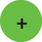 Low risk. 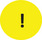 Some concerns. 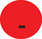 High risk. Outcomes: Maternal deaths, acute chest syndrome, pulmonary embolism, caesarean delivery, preterm delivery, and perinatal deaths. The risk of bias in vaso‐occlusive crises was considered high in both trials because the outcome assessor was the patient, and blinding of the participants was not feasible due to the nature of the intervention.

Since neither of the studies reported any maternal deaths, this variable was excluded from the meta‐analysis. Prophylactic transfusions may reduce the incidence of VOC in pregnant women with SCD, OR 0.197 (95% CI 0.08–0.49); low‐quality evidence from studies with low and uncertainty risk of bias. Prophylactic transfusion did not appear to reduce the incidence of ACS, OR 0.54 (95% CI 0.11–2.72), VTE, OR 1.99 (95% CI 0.16–24.64), caesarean delivery, OR 1.15 (95% CI 0.39–3.36), stillbirths, OR 1.79 (95% CI 0.36–8.93), and neonatal deaths, OR 2.741 (95% CI 0.24–31.13); low‐quality evidence from studies with low and uncertainty risk of bias. Prophylactic transfusions also did not appear to reduce preterm delivery, OR 0.82 (95% CI 0.04–16.02); very low‐quality evidence from studies with low and uncertain risk of bias. Despite a higher rate of transfusion in the prophylactic arm, there was no significant difference in transfusion reactions in this meta‐analysis, OR 1.71 (95% CI 0.68–4.27); very low‐quality evidence from studies with low and uncertain risk of bias. The random‐effects models of maternal and foetal outcomes are shown in Figure 2. Figure 3 presents the risk of bias assessment.

The studies were deemed homogeneous with respect to almost all the outcomes except for preterm delivery. Due to the small number of events, we could not explore causes of heterogeneity, use sensitivity analyses to assess the robustness of the synthesised results, or assess risk‐of‐bias due to missing results in the synthesis.

## 4 DISCUSSION

This meta‐analysis identified that prophylactic transfusion reduced VOC in pregnant women with SCD. However, the small sample size limited the ability to evaluate other outcomes comprehensively. Historically, women with SCD were discouraged from becoming pregnant due to the elevated frequency of complications, leading to high morbidity and mortality [14]. With improved care of children with SCD, more women reach reproductive age [3, 15]. Therefore, guidelines were developed to address the best practices for managing these patients. However, most recommendations suffer from low quality due to the absence of large, well‐designed RCTs and lack of funding.

Since there are currently no drugs recommended for SCD during pregnancy, transfusions represent the sole approach available for managing complications [15]. Transfusions can be given acutely with immediate benefits, such as increased oxygen‐carrying capacity [1]. Furthermore, the potential efficacy of prophylactic transfusion in pregnant women with SCD arises from its ability to improve SCD‐induced abnormalities [16]. Several retrospective studies published over the past decade have explored the use of prophylactic transfusions in managing pregnancy among SCD patients, showing promising results (Table 3) [17, 18, 19, 20, 21, 22]. Transfusion can correct anaemia and decrease the proportion of sickle erythrocytes, consequently reducing VOC. It also addresses the rise of oxygen consumption to match the metabolic needs of the foetus [3]. Furthermore, the use of exchange transfusion can lower viscosity, improving oxygen transportation [9, 23]. Despite the benefits, careful consideration is warranted to avoid indiscriminate transfusion, given the associated risks [1, 9, 24]. In a study of 99 DHTR cases, 31% of the patients were pregnant women [25], highlighting the need of a robust evidence of the benefits of transfusion in this population.

**TABLE 3:** Overview of retrospective studies on prophylactic transfusions during pregnancy in sickle cell disease patients (2014–2024).

		Pregnancies				
Authors	Title	Total	Prophylactic transfusions	Year of publication	Benefits	Risks
Anil Ananthaneni, Sarah Jonesa, Mohamed Ghoweba, Vishwa Granta, Kenna Leethyc, Taras Benzara, Samip Mastera, Richard Mansoura, Poornima Ramadas	Impact of scheduled partial exchange transfusions on outcomes in pregnant patients with severe sickle cell disease: a retrospective study	47	14	2024	Fewer admissions for pain crises (*p* = 0.032), higher gestational age at delivery (*p* = 0.007), and a lower incidence of neonatal intensive care admissions (p = 0.011)	Alloimmunisation: Nil Other reactions: Not addressed
Sobczyk O, Gottardi E, Lefebvre M, Canouï‐Poitrine F, Jebali A, De Luna G, Pirenne F, Redel D, Galacteros F, Boutin E, Bartolucci P, Haddad B, Habibi A, Lecarpentier E.	Evaluation of a prophylactic transfusion program on obstetric outcomes in pregnant women with sickle cell disease: A single centre retrospective cohort study	224	148	2023	Reduced preterm birth prior to 34 weeks (*p* = 0.001), vaso‐occlusive crisis (*p* < 0.001), and acute chest syndrome (*p* = 0.04)	Alloimmunisation Similar frequency between groups. Haemolytic transfusion reactions: Similar frequency between groups
Yılmaz Baran Ş, Kozanoğlu İ, Korur A, Doğan Durdağ G, Kalaycı H, Alemdaroğlu S, Asma S, Kılıçdağ EB, Boğa C.	Role of prophylactic and therapeutic red blood cell exchange in pregnancy with sickle cell disease: Maternal and perinatal outcomes	46	27	2021	Reduced painful crises (*p* = 0.001), preeclampsia (*p* = 0.024) and preterm birth (*p* = 0.027)	Alloimmunisation: One patient Mild hypocalcaemia: Three patients
Zamané H, Sanou F, Kiemtoré S, Kain DP, Sawadogo AK, Bonané‐Thiéba B.	Transfusion practices in the care of pregnant women with sickle cell disease in Ouagadougou	164	53	2019	Reduced maternal complications in the third trimester (*p* < 0.001), postpartum maternal morbidity (*p* = 0.003) and postpartum maternal mortality (*p* < 0.009)	Not addressed
Benites BD, Benevides TC, Valente IS, Marques JF Jr, Gilli SC, Saad ST.	The effects of exchange transfusion for prevention of complications during pregnancy of sickle haemoglobin C (SC) disease patients	24	10	2016	Increased gestational age at birth (38.7 weeks in the transfusion group vs. 34.4 weeks (*p* = 0.037).	Alloimmunisation: Nil Other reactions: Not addressed
Asma S, Kozanoglu I, Tarım E, Sarıturk C, Gereklioglu C, Akdeniz A, Kasar M, Turgut NH, Yeral M, Kandemir F, Boga C, Ozdogu H.	Prophylactic red blood cell exchange may be beneficial in the management of sickle cell disease in pregnancy.	37	24	2015	Reduced maternal complications (*p* = 0.042) and mortality (*p* = 0.011)	Alloimmunisation: Four patients

*Note*: We included only studies that compared pregnancy outcomes between prophylactic transfusion and routine care, with at least 10 patients receiving prophylactic transfusions during pregnancy.

A previous meta‐analysis with 12 studies, combining RCTs, non‐RCTs and observation studies, demonstrated that prophylactic transfusion was associated with a reduction in maternal and foetal/neonatal morbidity and mortality. The studies included in the meta‐analysis were considered to have moderate to high risk of bias [26]. A subsequent meta‐analysis, comprising solely of RCTs and published in 2016, was unable to address this issue due to the limited availability of RCT [27]. Despite a lack of robust evidence, many centres introduced the use of prophylactic transfusion after case‐by‐case discussion [28].

Pregnant women with SCD suffer from increasing RBC sickling, along with other physiological changes observed in all pregnancies, such as an increase in metabolic demand and leucocyte count [29]. These alterations lead to a higher risk of VOC, which is a frequent cause of poor quality of life and hospitalisations [29]. Reducing VOC may mitigate other less common complications, such as VTE and ACS, as discussed later. As the patient is the outcome assessor for VOC, and due to the nature of the intervention, the patient is not blinded, and we may encounter a higher risk of bias for this outcome.

ACS presents a potentially life‐threatening situation, demanding emergent and specialised care [1, 30]. In pregnancy, ACS ranks as the second most frequent reason for hospitalisations and the primary cause of mortality [29]. The elevated incidence of ACS during pregnancy can be attributed to the increased risk of infections, VTE, and VOC, which are known precipitating factors [14, 29, 30]. In a previous systematic review, the combined prevalence of ACS and pneumonia in pregnant women with SCD was 6.46% (95% CI, 4.66–8.25). The analysis found no significant difference in the prevalence of ACS/pneumonia between the HbSS and HbSC genotypes (relative risk, 1.42; 95% CI, 0.90–2.23) [31]. Due to the small number of patients included in our meta‐analysis, it was not possible to evaluate accurately the role of transfusion as a preventive measure for this outcome.

Both pregnancy and SCD individually pose a risk of VTE [7]. During pregnancy, there's a gradual development of a hypercoagulable state characterised by elevated levels of clotting factors and reduction in the activity of anticoagulant proteins. Moreover, the risk is exacerbated by venous stasis, due to decreased venous tone and compression of the inferior vena cava and iliac veins by the uterus [32]. Furthermore, SCD is acknowledged as a prothrombotic condition due to heightened platelet function, impaired fibrinolysis, and continuous coagulation activation [33]. The estimated prevalence of pulmonary embolism in SCD pregnant women was reported as 105 per 10,000 (95% CI, 65–170). This is nearly an 8­fold increased risk in women with SCD (relative risk, 7.74; 95% CI, 4.65–12.89) compared to pregnant women without SCD [31]. Due to the limited number of patients included in this meta‐analysis, it was not feasible to assess the impact of prophylactic transfusion on this complication. Regardless, hospitalizations increase the risk of thrombosis [33], with VOC being the primary cause of hospital admissions in SCD pregnancies [29]. Therefore, reducing VOC may positively impact the occurrence of VTE. Furthermore, exchange transfusion can theoretically reduce the risk of VTE, since it not only diminishes blood viscosity but also lowers the counts of leucocytes, neutrophils, and platelets, along with reducing plasma levels of vascular cell adhesion molecule‐1 [34, 35].

Women of African descent are three to four times more likely to have a very early preterm birth than women from other racial or ethnic groups [36]. Furthermore, infants born to mothers with SCD face heightened risks of IUGR, low birth weight, and prematurity. These data remain consistent across low‐ and high‐income countries [5, 7]. The persistent inflammation observed in SCD, with sickling and endothelial damage, appears to be a risk factor for pre‐eclampsia, potentially leading to restricted foetal growth and an increased risk of preterm birth [3, 7]. Also, anaemia affects foetal growth both in the general population and among women with SCD [3]. Additionally, vaso‐occlusion and vasculopathy may influence placental health [3]. Therefore, there is a potential role of early exchange–transfusion in improving the infant birthweights of women with SCD [16]. Koshy did not evaluate pre‐eclampsia [13], and we did not include this outcome in this meta‐analysis. Despite the theoretical benefit, Malinowski's meta‐analysis did not observe any effect of prophylactic transfusion on pre‐eclampsia and foetal growth [26]. The authors attributed this to the considerable variation observed among studies regarding the initiation timing of transfusions, as early aberrant placentation is often the underlying pathophysiological mechanism for these conditions [26]. The small number of patients included in our meta‐analysis, along with the late intervention in Koshy study, can also justify the absence of effect of prophylactic transfusion in foetal development. Furthermore, preterm delivery is common among patients with HbSS [8], and prophylactic transfusion may reduce the risk of this complication [22]. In this meta‐analysis, there was heterogeneity regarding this outcome between the included studies. Preterm delivery was more frequent in the intervention arm in Koshy. Koshy included multiple gestations, that carry a substantial risk of premature delivery with almost 60% of twins born preterm [36]. Additionally, in Koshy's paper, a larger proportion of patients in the intervention group reported alcohol consumption during pregnancy, but the exact level of alcohol intake could not be accurately assessed, as it was only noted as exceeding two alcoholic beverages per week [13]. Furthermore, while heavy alcohol consumption is associated with an increased risk of preterm birth, mild to moderate alcohol use is generally not considered a significant risk factor for this outcome [36]. This paper did not present any information regarding past premature deliveries, which is one of the risks factors for a subsequent premature delivery [36]. We could not provide subgroup analysis to address the heterogeneity of this outcome since we did not have the primary data from Koshy, and we had a very small population in each arm. There were more caesarean deliveries in the Oteng‐Ntim study, most of which were elective. The frequency of elective caesarean was unavailable in the Koshy study; hence it was impossible to evaluate this outcome thoroughly.

In the Koshy study, while the groups exhibited close similarity in most of baseline characteristics, the intervention group notably showed a higher incidence of two significant risk factors for perinatal mortality: seven patients compared to one for previous perinatal mortality, and three patients compared to one for multiple gestations [13]. There were no perinatal deaths in Oteng‐Ntim, likely due to the improved care in recent years [3, 4]. Due to the small number of patients, we could not provide a strong evidence of the impact of prophylactic transfusion on perinatal mortality.

It is important to highlight that the risk of bias for the Koshy study was uncertain for most of the outcomes as the trial reports contained little methodological description and only the manuscript with the results was published and available [13]. The risk of bias for the Oteng‐Ntim RCT was considered low for most of the outcomes. The protocol, the statistical analysis plan, and the results were all accessible for review [11, 23, 37]. Concerning VOC, since the outcome assessor is the patient, and blinding of the participants was not possible due to the nature of the intervention, the risk of bias in measurement of this outcome was considered high for both trials.

There are some important differences between the two studies included in this meta‐analysis. Firstly, the Koshy study included only SS patients, while the Oteng‐Ntim included other SCD genotypes. Secondly, the timing for commencing prophylactic transfusion differed: Koshy included patients up to 28 weeks of gestation, whereas Oteng‐Ntim, up to 18 weeks. This early intervention may lead to better outcomes. Furthermore, transfusion medicine improved significantly over the years and automated‐exchange, used in Oteng‐Ntim, is much more effective than top‐up and partial manual‐exchange (used in Koshy) to achieve lower HbS targets in shorter periods of time [38]. HbS levels below 30% can be attained in a single procedure lasting less than 2 h [9]. Altogether, we can safely assume that the women in Koshy achieved their HbS target later than in Oteng‐Ntim. Erythrocytapheresis is now widely accessible in middle‐ to upper‐income countries. Despite potential risks associated with venous access and using citrate, it is a safe procedure and the preferred method for exchange in SCD [9, 16, 38]. Similar challenges were observed in Malinowiski's meta‐analysis. There was considerable variability in transfusion protocols among the studies, with differences noted not only in the timing and modalities employed, but also in transfusion targets and subsequent triggers for transfusion across studies [26].

Another factor that differentiates the two studies is the use of Rh and K‐matched RBC units in Oteng‐Ntim. This approach is largely used in middle‐ and high‐income countries to avoid RBC alloimmunization in SCD patients [24, 39]. Despite the use of erythrocytapheresis in Oteng‐Ntim and the need for more RBC units per procedure, this approach is considered at least equivalent to manual exchange or top‐up transfusion regarding alloimmunization if Rh and K RBC units are used [16]. Furthermore, on‐demand transfusion is typically administered in urgent situations, whereas prophylactic transfusion offers a non‐urgent, planned strategy [26]. It is easier to provide extended phenotyped or genotyped‐matched RBC for planned transfusions, and this strategy can reduce further the frequency of alloimmunization [40]. Also, alloimmunisation risk increases when transfusions are administered in acute settings [9, 24, 28, 39, 41].

In addition to the limitations discussed earlier, several other important points warrant attention. First, there is a considerable time gap between the two publications included in this review, during which standards of care have evolved significantly. Second, the combined sample size across both studies is relatively small, with only 54 women in the prophylactic arm and 53 in the control arm, which is limited given the broader population of women with SCD. This small sample size poses a significant constraint on drawing robust conclusions. Furthermore, the quality of evidence was downgraded in multiple areas and rated as low or very low, suggesting a high likelihood that the true effect may substantially differ from the estimated effect. Additionally, due to the small sample size and the low frequency of events, the range of effect estimates for some variables is very wide.

This is the first systematic review focusing solely on RCTs following the publication of the TAPS2 trial, whereas the prior Cochrane review [27] included only the Koshy study. Despite some centres adopting prophylactic transfusion as standard care [14], its use remains inconsistent due to the lack of robust RCT evidence and concerns about transfusion risks. The scarcity of RCTs, evidenced by only two studies included in this meta‐analysis, highlights a critical gap in evidence‐based care.

In conclusion, this meta‐analysis suggests that prophylactic transfusion may improve outcomes for pregnant women with SCD by reducing VOC without a significant increase in transfusion reactions. Given that VOC is associated with a high risk of hospitalisation and complications such as ACS and VTE, reducing VOC could potentially mitigate other risks for both the mother and baby. Equally, this meta‐analysis highlights the paucity of evidence to guide the use of transfusion in this cohort of women, who face significant morbidity during pregnancy. Multicentre RCTs with robust designs and larger sample sizes are needed to comprehensively evaluate the clinical impact and associated risks of prophylactic transfusion in this patient population. Addressing this gap is crucial to provide stronger evidence to guide and standardise care for this high‐risk group.

## AUTHOR CONTRIBUTIONS

*Concept and design*: Eugene Oteng‐Ntim, Denise Menezes Brunetta and Paul T. Seed. *Acquisition, analysis, or interpretation of data*: Eugene Oteng‐Ntim, Denise Menezes Brunetta, Evangelia Vlachodimitropoulou and Nita Prasannan. *Drafting of the manuscript*: Denise Menezes Brunetta, Evangelia Vlachodimitropoulou, Nita Prasannan and Eugene Oteng‐Ntim. *Critical revision of the manuscript for important intellectual content*: Denise Menezes Brunetta, Evangelia Vlachodimitropoulou, Nita Prasannan, Eugene Oteng‐Ntim and Paul T. Seed. *Statistical analysis*: Denise Menezes Brunetta and Paul T. Seed.

## CONFLICT OF INTEREST STATEMENT

The authors declare no conflicts of interest.

## FUNDING INFORMATION

This paper received no specific grant from any funding agency in the public, commercial, or not‐for‐profit sectors.

## ETHICS STATEMENT

The authors have confirmed ethical approval statement is not needed for this submission.

## PATIENT CONSENT STATEMENT

The authors have confirmed patient consent statement is not needed for this submission.

## CLINICAL TRIAL REGISTRATION

The authors have confirmed clinical trial registration is not needed for this submission.

## Data Availability

The data that support the findings of this study are available upon reasonable request from the corresponding author, Eugene Oteng‐Ntim (Eugene.Oteng‐Ntim@gstt.nhs.uk).
